# Robotic Extrusion of Algae‐Laden Hydrogels for Large‐Scale Applications

**DOI:** 10.1002/gch2.201900064

**Published:** 2019-11-11

**Authors:** Shneel Malik, Julie Hagopian, Sanika Mohite, Cao Lintong, Laura Stoffels, Sofoklis Giannakopoulos, Richard Beckett, Christopher Leung, Javier Ruiz, Marcos Cruz, Brenda Parker

**Affiliations:** ^1^ Bartlett School of Architecture University College London London WC1E 6BT UK; ^2^ Department of Biochemical Engineering Bernard Katz Building University College London London WC1H 0AH UK; ^3^ Institute of Structural and Molecular Biology University College London London WC1E 6BT UK; ^4^ M. Alexander 19 Athens 15773 Greece

**Keywords:** additive manufacturing, hydrogels, immobilization, microalgae, photosynthetic, robotic extrusion

## Abstract

A bioprinting technique for large‐scale, custom‐printed immobilization of microalgae is developed for potential applications within architecture and the built environment. Alginate‐based hydrogels with various rheology modifying polymers and varying water percentages are characterized to establish a window of operation suitable for layer‐by‐layer deposition on a large scale. Hydrogels formulated with methylcellulose and carrageenan, with water percentages ranging from 80% to 92.5%, demonstrate a dominant viscoelastic solid–like property with *G′* > *G″* and a low phase angle, making them the most suitable for extrusion‐based printing. A custom multimaterial pneumatic extrusion system is developed to be attached on the end effector of an industrial multiaxis robot arm, allowing precision‐based numerically controlled layered deposition of the viscous hydrogel. The relationship between the various printing parameters, namely air pressure, material viscosity, viscoelasticity, feed rate, printing distance, nozzle diameter, and the speed of printing, are characterized to achieve the desired resolution of the component. Printed prototypes are postcured in CaCl_2_ via crosslinking. Biocompatibility tests show that cells can survive for 21 days after printing the constructs. To demonstrate the methodology for scale‐up, a 1000 × 500 mm fibrous hydrogel panel is additively deposited with 3 different hydrogels with varying water percentages.

## Graphical abstract

Algae‐laden hydrogels are 3D‐printed on a large scale through a custom‐built robotic extrusion system. Rheological characterization of alginate hydrogels identified physiochemical and biological properties suitable for 3D construction. Algal cells immobilized in CaCl_2_ survived extrusion, demonstrating the biocompatibility of the material and printing process. This work demonstrates potential for the design, fabrication, and scale‐up of photosynthetic membranes in architecture.

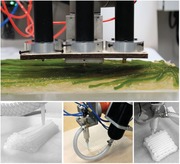

## 1 Introduction

Additive manufacturing (AM) is being increasingly adopted across a wide range of fields for applications ranging from the nano‐ to micro‐ and macroscales. The field of tissue regeneration 3D prints high resolution biocompatible scaffolds of a few hundred micrometers for cell cultures with the aim of replacing or reconstructing tissue within the human body.^1^, ^2^ Whereas on the macroscale, 3D printing is widely utilized for general prototyping, and is now beginning to be used to achieve performance‐specific and cost‐effective building construction or manufacturing of prefabricated components.^3^ One of the major advantages of AM is the ability to allow for the homogenous construction of hybrid components from heterogeneous materials, in a layer‐by‐layer manner.

With the goal of tackling the extreme environmental problems of our cities, research into living architecture aims to incorporate biological elements within structures, termed here as biohybrid. There is a growing movement to develop new methods for creating biohybrid structures that could potentially increase vegetation and green cover onto building envelopes and rooftops. These biohybrid structures may improve the environmental air quality by lowering atmospheric CO_2_ through photosynthesis, performing energy generation via technologies such as biophotovoltaics,^4^ or creating an ecological habitat with advantages such as storm water management.^5^

Previous attempts at biohybrid structures included photobioreactors that were applied onto the building envelope. The BIQ House in Hamburg deployed large‐scale flat photobioreactors on the building facade, in order to cultivate large amounts of microalgae as part of an integrated strategy to generate biomass and bioenergy.^6^ On the other hand, bioreceptive walls are a potential strategy to avoid the cycles of high maintenance which currently limits the design and fabrication towards ready scale‐up of green walls.^7^ More recently, two patents associated to algae–façade systems have been submitted and their future potentials as sustainable energy generating façade alternatives are being tested.^8^, ^9^, ^10^ All three façade systems are designed as substitutions to the conventional curtain wall using large amounts of glass and metal fittings as prime components for module production. By contrast, emerging forms of interdisciplinary research are using existing techniques of top‐down design and fabrication to construct artificial biological processes from the bottom‐up. Therefore, we hypothesize that in the future, these self‐regulated biohybrid systems could be custom fabricated using efficient techniques of AM from biocompatible materials over varying range of scales. Therefore, as a workflow demonstrator, this research builds on the emerging discourse of algae–bioreactor façade systems as biohybrid structures for large‐scale applications.^11^

Immobilization of whole cells represents an alternative to cultivation of biomass within photobioreactor systems. Typically, immobilization makes use of packed bed reactors, however it has proved challenging to create a homogeneous environment for cell culture. The use of additive manufacturing has enabled a priori design of packed bed systems for immobilization. This has previously been demonstrated in other biochemical engineering applications where greater control over fluid flow has led to gains in chromatography performance.^12^ Previously techniques such as adsorption, entrapment of whole cells in porous polymers or microcapsules, covalent coupling, and self‐adhesive attachment of cells onto the surfaces of solid supports or organic/inorganic support matrices have been explored for cell immobilization.^13^, ^14^ However, the major challenge remains in creating more efficient and stronger immobilization matrices, wherein the 3D gel lattices are optimized for high surface area‐to‐volume ratios, directly affecting the current low growth rates as compared to their free‐living counterparts. Hydrogels have been used in previous studies to immobilize microalgae.^13^, ^14^, ^15^, ^16^ Most recently, the successful 3D bioprinting of cell‐friendly hydrogel constructs, including fabrication of photosynthetic algae‐laden hydrogel scaffolds^17^, ^18^ at laboratory scale using proprietary equipment has been demonstrated.^19^, ^20^

Hydrogels are water‐based polymeric materials that form physical bonds, hydrogen bonds, or chemical crosslinks via ionic and hydrophobic interactions.^1^, ^21^, ^22^ They provide both mechanical and environmental support compatible with cell proliferation.^23^, ^24^ Hydrogels demonstrate properties of selective permeability,^21^ permitting the entrapped cells to perform functions such as photosynthesis through mass transfer of nutrients and diffusion of carbon dioxide.^16^ At present, there exists a range of industrial 3D printers capable of fabricating small‐scale hydrogel scaffolds, ranging from 100 nm to 50 mm for biomedical applications.^2^ Polysaccharide‐based hydrogels have the potential to be biodegradable and recyclable. Recent research demonstrated the possibility of AM large‐scale functionally graded hydrogel composites via a multichamber extrusion system. The prototypes were presented as biodegradable‐composite objects as they were primarily composed of chitosan and sodium alginate with other organic aggregates which could be chemically stabilized or dissolved in water and recycled.^25^, ^26^, ^27^

**Figure 1:**
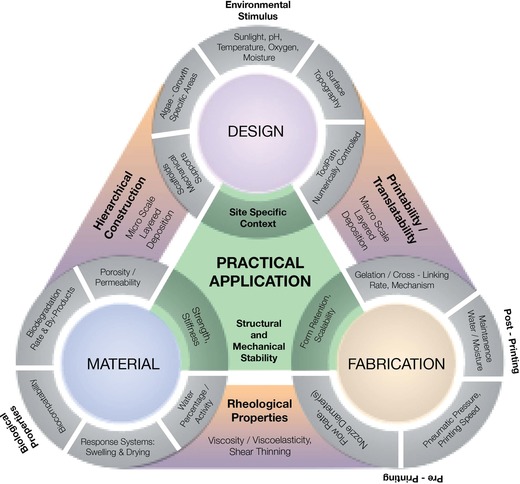
Schematic illustrating the interdependency of the four prime parameters – Material (Section 2.1), Fabrication (Section 2.3 and Experimental Section (*Robotic Fabrication—Hardware and Printing Setup* and *Robotic Fabrication—Software*)), Design (Section 2.4), and Application (Section 2.5); along with their individual characteristics that together inform the development of a biocompatible system.

As illustrated in **Figure** **1**, fabrication of hydrogels on a large scale of 500–1000 mm and above requires bespoke manufacturing methods contingent upon an interplay of factors ranging from the micro‐ to the macroscale. The properties of the hydrogel are defined by its formulation, influencing the deposition along with the mechanical integrity of the structure printed. Ideally, the properties of a biocompatible hydrogel for large‐scale AM should exhibit optimum viscoelasticity with a dominant solid‐like property, allowing the material to flow under pressure eventually recovering and retaining its deposited form without affecting the compatibility of the cells. The layers thus extruded should also exhibit a high mechanical strength with the ability to hold its shape, while additional layers are deposited and crosslinking via postcuring is performed, preventing deformation or collapse of the structure. This should be complimented by a reduced die swell effect which prevents the expansion of the strand diameter upon extrusion through an orifice under pressure. Mathematically, the die swell effect, *D* = *D*_ex_/*D*_o_ wherein an extruded strand with a cross‐section *D*_ex_ is observed to be greater than the die cross‐section *D*_o_.^28^ Primarily, the exploration of this workflow is based on achieving large‐scale biocompatible structures, therefore, the hydrogels on a microscale shall allow cell proliferation while also preventing cellular damage from any shear stress incurred through the extrusion process. The hydrogel further exhibits properties such as drying and swelling, in the absence or presence of additional moisture in its surrounding, resulting in deformation through shrinkage and warping. This offers design challenges specific to AM, where the print geometry is computationally generated optimizing various printing parameters according to the altering behavior of the hydrogel, with respect to its water content in order to be able to fabricate a hierarchically constructed, mechanically stable, and a biologically active structure.

Here, we develop a large‐scale pneumatically driven robotic extrusion technique that demonstrates the layer‐by‐layer fabrication of algae‐laden hydrogels. In order to establish the material compatibility for large‐scale printing, we have rheologically characterized hydrogel behavior under shear stress and assessed its relative printability. We used computationally simulated design techniques to generate hierarchical fibrous patterns to test the resolution of the printing system. The printed panels were maintained postcuring to observe the behavior of microalgal cells and the longevity of the biohybrid material.

Such an approach demonstrates the ability to develop materials with biological properties suitable for large‐scale manufacturing.^29^ This work could have applications in the areas of bioremediation that utilize the absorptive ability of algae cells to capture nutrients, heavy metals from wastewater; or in creating secondary products such as biohydrogen that utilize the photosynthetic activity of immobilized algae cells.

## 2 Results and Discussion

### 2.1 Material Characterization of Alginate‐Based Hydrogels

For a pneumatic‐based extrusion system, there are two characteristics that make a gel formulation suitable for printing. First, the hydrogel should possess a low transitional viscosity that allows the material to flow through a nozzle under sufficient applied pressure; while also demonstrating a viscoelastic solid–like property that allows the material to retain its form postextrusion.^30^, ^31^ Rheologically, the hydrogel should remain within a low phase angle, i.e., ratio between *G′* and *G″*, significant of the hydrogel samples' solid‐like property at low frequency but acquires a more liquid‐like property under high frequency, or more specifically high pressure. Yield stress and shear strain are other important factors in characterizing 3D‐printable materials.

Initial rheological measurements confirmed the inverse relationship between the hydrogels' water content and their relative viscosity. For alginate‐based hydrogel samples with varying water percentages, sample 01 with the maximum water percentage exhibited the least viscosity and sample 04 with the least water percentage showed the maximum viscosity. For samples with a uniform water percentage and varying rheology modifiers, the alginate–carrageenan‐based hydrogel (sample 05) showed the highest viscosity whereas, curran‐based hydrogel (sample 06) showed the lowest viscosity.

The amplitude–strain sweep test highlighted the distinct linear viscoelastic (LVE) (the time‐dependent behavior of polymers between the stress and strain) limit of sample 05, demonstrating the hydrogel's requirement of an initial pressure greater than 1.5–2.5 bar in order to attain a fluid‐like behavior. Other samples showed constant LVE limit, however their values increased or decreased as a function of their respective viscosities.

**Figure 2:**
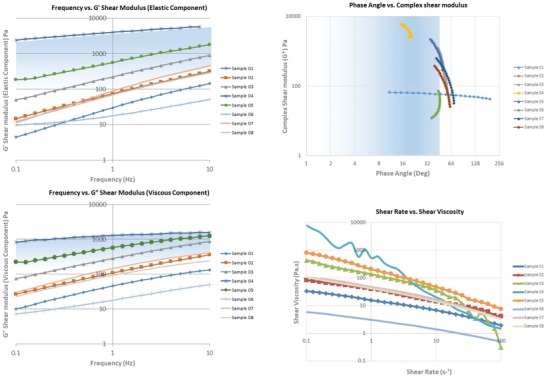
Rheological characterization of printable hydrogels – the shaded region shows the optimum range of parameters a hydrogel should exhibit in order to 3D print with. The blue shaded region shows higher degree of printability with form retention, whereas the lighter region shows comparatively less printable hydrogels.

The frequency sweep tests determined the dominant property of the gel as either solid‐like or liquid‐like. Samples 02 and 03 showed the most robust properties, while samples 04–06 showed a dominant elastic component (*G′* > *G″*), allowing the hydrogel to recover its initial form when applied shear stress was removed. Further, the notable degree of dependency of phase angle with frequency highlighted the hydrogels' ability to behave as a viscoelastic liquid, under certain shear conditions. Results are summarized in **Table** **1** and **Figure** **2**.

**Table 1:** Summary of the rheological properties of all the samples tested

S. No.	Sample	Water percentage (by weight)	Relation between *G′* and *G″*	Dominant property	Phase angle [Deg, °]	Extrusion pressure [bar]
01.	50 mL water + 1.5 g alginate	97%	*G″* > *G′*	Viscoelastic fluid	56	1.5
02.	75 mL water + 1.5 g alginate + 4.5 g methylcellulose	92.5%	*G″* > *G′*	Viscoelastic fluid	55	1.8
03.	50 mL water + 1.5 g alginate + 4.5 g methylcellulose	89.2%	*G″* > *G′*	Viscoelastic fluid	51.2	2
04.	25 mL water + 1.5 g alginate + 4.5 g methylcellulose	80.6%	*G′* > *G″*	Viscoelastic solid	18.2	3–4
05.	75 mL water + 1.5 g alginate + 4.5 g Carrageenan	92.5%	*G′* > *G″*	Viscoelastic solid	44.4	3
06.	75 mL water + 1.5 g alginate + 4.5 g Curran	92.5%	*G′* > *G″*	Viscoelastic solid	43.5	2
07.	75 mL water + 1.5 g alginate + 4.5 g Laponite	92.5%	*G″* > *G′*	Viscoelastic fluid	57.1	1.2
08.	75 mL water + 1.5 g alginate + 4.5 mL Ludox	92.5%	*G″* > *G′*	Viscoelastic fluid	52.3	1.2

The flow curve tests demonstrated that the lower water concentration samples showed high resistance to flow. The varying flow rates of samples 03–05 showed the change in material behavior beyond its threshold shear stress limit. The steep reduction in the apparent viscosity of sample 04 showed that the sample can flow but under extremely high shear stress; while showing a Bingham plastic‐like behavior. Therefore, samples 03–05 could be extruded by applying high shear rate during injection, and these types of gels could self‐heal shortly after applied shear stress was removed, owing to the thixotropic property of the material.^32^

All alginate‐based hydrogel samples demonstrated a shear thinning behavior, which reduced the die swell problem, allowing the filament to emerge smoothly.^33^ The die swell effect was associated with the viscoelastic nature of polymer melts, wherein the material upon extrusion swelled when forced through an orifice impacting the profile deposited onto the printing platform. Notably, the addition of monomers added stiffness to the gels, proportionally affecting the shear stress required for the fluid to flow as a function of shear rate.

As per the data collected highlighted in Figure 2, it was desirable for a hydrogel to have an LVE limit within the region enclosed by samples 04, 05, 03, and 07, as this allowed the shear modulus to be independent to the frequency up to a certain limit. Operating within this limit retained solid‐like mechanical properties while allowing the hydrogel to flow under pressure. Further, alginate‐based hydrogels with *G′* > *G″* demonstrating a dominant viscoelastic solid‐like property were preferred, allowing the hydrogel to flow through the nozzle to self‐heal or gain its initial state postextrusion. Therefore, samples 03 and 04 with the same rheology modifier – methylcellulose was selected for conducting further 3D printing tests. Preliminary extrusion was also conducted on sample 02 in order to verify and establish the rheological findings with the practical setup.

### 2.2 Cell Viability Test

#### 2.2.1 Macroscale

**Figure 3:**
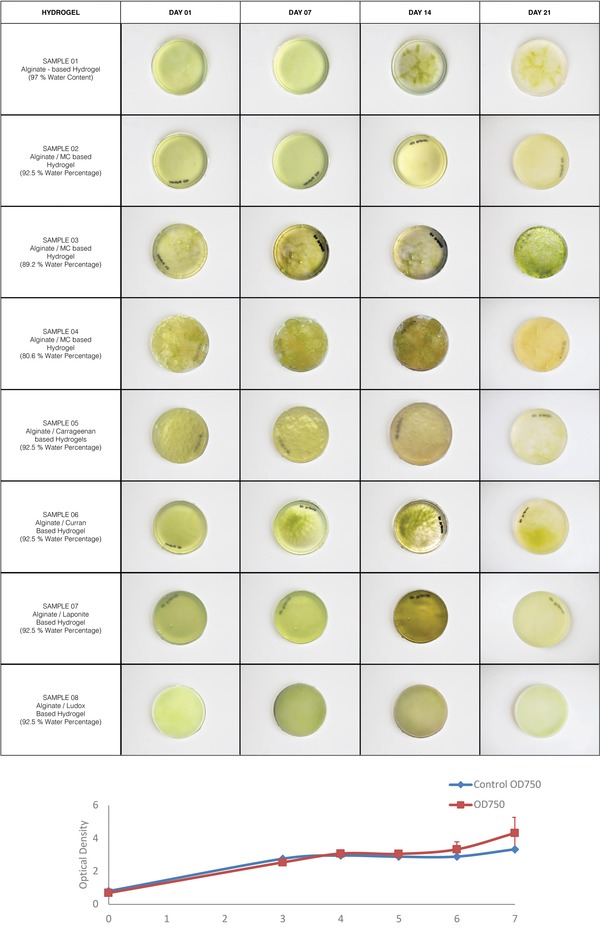
a) Cellular viability tests are conducted on a macroscale. Petri dishes were supplied with 5 mL TAP media every 7 days in order to maintain the growth of algae cells. b) Growth curve of *C. sorokiniana*, when sheared under a rate of 18 000 rpm for 20 s. Post which, the algae solution was cultured in TAP medium to grow for 7 days. OD was tested on days 1, 3, 4, 5, 6, and 7.

Two tests were conducted to assess the suitability of hydrogel material and the effect of the deposition process toward cellular growth. **Figure** **3**a shows the growth of *Chlorella sorokiniana* in all of the hydrogel samples 01–08 over a period of 21 days. Visible growth was observed (change in coloration) in all samples during the first 7–10 days. Hydrogel sample 03 (89.2% water percentage) showed the maximum visible cellular growth. This could be primarily due to an optimum combination between the water content, pore size, and the biocompatibility of the polymer methylcellulose toward the algae cells. Interestingly, the cellular growth rate is not directly linked with the increase in water content within the hydrogels, otherwise samples 01 and 02 with the highest water content should have shown the maximum growth rate. Observations show that crosslinking with CaCl_2_ forms an extracellular matrix suitable for cellular growth, which is dependent upon the hydrogels' water content.^18^ This can also be read in conjunction with the surface‐area‐to‐volume ratio within the hydrogel matrix which allows sufficient porosity, in terms of light and nutrient supply for algae cells to perform photosynthesis and multiply.^34^ Therefore, a hydrogel with either the maximum or the minimum water percentage such as samples 01, 02, and 04 did not show high biocompatibility. Samples 05–08 were tests of cellular compatibility of the different rheology modifiers. While all the monomers show algae survivability for the first 7–14 days – carrageenan in addition to methylcellulose exhibited the highest compatibility. Sample 03 continued to show cellular growth until day 21, while the rest of the samples turned pale and discolored (Figure 3a).

#### 2.2.2 Microscale

The algae‐laden hydrogel passes through varying levels of shear within the workflow, from manually stirring the cell culture with the hydrogel prior to printing, to loading the hydrogel in the cartridge, and its forced extrusion through the dispensing nozzle's small orifice. While survivability tests of algal cells upon 3D plotting have been conducted before,^17^, ^18^ it was crucial to test the cell viability within this research as the scale of printing and therefore its various parameters including shear stress and pressure at the multiple stages of the workflow have increased significantly. The shear stress experienced by the cells under high pressure could potentially harm the cells upon extrusion. However, the growth curve presented in Figure 3b shows that 18 000 rpm shear had no significant effect on the growth of *C. sorokiniana* cells. The growth rate of the algal cells under shear was similar to the growth of algae without shearing. Combined with the protective effect of the hydrogel matrix, this supports our hypothesis that the process of pneumatic extrusion through a nozzle does not affect the cellular growth and viability of algae cells.^35^

### 2.3 Printability

#### 2.3.1 Mathematical Model between Line Width, Pressure, and Printing Speed

The flow rate *Q* through the nozzle can be mathematically constructed using the power law model.^36^ As per which, the flow rate *Q* is equal to (*πd* ^2^/4) × *S*, where *d* refers to the printed strand diameter and *S* to the speed of the motion platform. Demonstrating that the flow rate *Q* is directly proportional to the speed *S*, affecting the line width *d* of the deposited hydrogel strand. We assumed the flow rate to be constant from start to the end point, and that the strand was printed in a continuous manner.

Further, the maximum shear stress experienced by the material during capillary flow (material forced through a restricted geometry) is directly proportional to the apparent viscosity of the material η.

**Figure 4:**
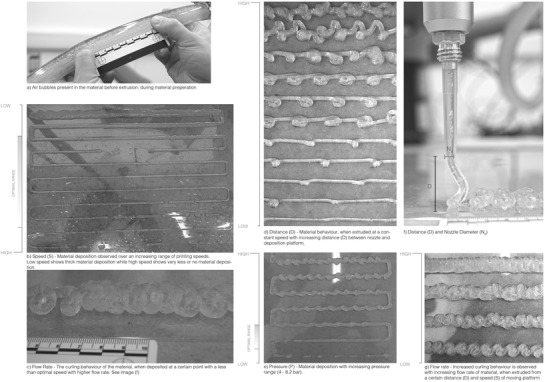
a–g) Initial printing tests to establish optimum relationships between – nozzle diameter (*N*_d_), flow rate *Q* (relative to pressure “*P*”), printing speed (*S*) of moving platform, distance “*D*” between nozzle and deposition platform. These tests are conducted with alginate–methylcellulose hydrogel with 89.2% water content (Table 1, sample 03).

**Figure 5:**
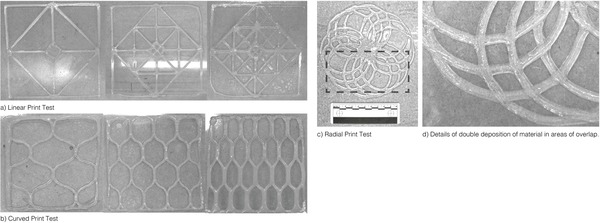
a–d) Pattern‐based printing tests – pneumatic extrusion tests are conducted on simple patterns and geometries to observe the continuity and accuracy of printing. Points of overlap are also tested for the hydrogels ability to retain form upon layered extrusion, accompanied by density and adjacency of printing lines and layers. These tests are conducted with alginate–methylcellulose hydrogel with 89.2% water content (Table 1, Sample 03).

Mathematically, the resistance of a sample against flow η is the ratio between the maximum stress in the capillary flow τ and the shear rate γ. Shear rate γ = (32/π) × (*Q*/*d* ^3^), highlighting its interdependency on flow rate *Q*. Therefore, the volumetric flow rate *Q* is increased as pressure applied increased; likewise the shear rate experienced by algae cells is also increased linearly.^37^, ^38^, ^39^ The relations are read in conjunction with the initial printing tests illustrated in **Figures** **4** and **5**.

#### 2.3.2 Line Printing

Alginate‐based hydrogels with low viscosities, as seen in sample 02, were able to flow under low pressure. However, they spilled and lost their morphological stability postextrusion. Samples 03 and 04 with higher viscosity, demonstrated higher resistance to flow and required a constant pressure gradient. In order to keep the pressure within achievable limits, the speed of the moving platform had to be reduced to print consistent lines. These samples depicted a higher structural integrity, retaining subsequent layers of print.

Furthermore, an optimal distance *N*_d_ between the nozzle and the deposition platform (*Z*‐direction) needed to be established specific to the flow rate and speed, preventing the material from curling/warping/breaking at certain points. For instance, if *N*_d_ was too high and its relative speed too slow, as shown in Figure 4d,f, the hydrogel strands would curl. This was an important parameter that determined the line width deposited over the printing platform. Figure 4b shows the change in deposition behavior with increasing speed, while all other parameters are kept constant. As discussed above, the speed is directly proportional to the flow rate which is inversely proportional to the viscosity of the hydrogel, Figure 4c. Therefore, depending upon the sample rheology, viscosity, and the relative nozzle diameter, the line width expanded postextrusion. If the hydrogel was less viscous and the nozzle diameter 4–7 mm, the hydrogel strands deposited would be thicker, eventually spilling into a nondefined geometry. The extent of this could be mitigated by spraying 100 × 10^−3^ m calcium chloride solution over the deposited line in order to retain the extruded hydrogel via crosslinking.

Adjacency tests were conducted to illustrate the rate of diffusion between two consecutive printed lines as a function of the hydrogels viscosity. The higher the material viscosity used, the lower the diffusion rate of the hydrogel with line distances as less as 2 mm. However, after the grid was immobilized and left under observation at local room temperature and pressure conditions, the lines printed closer than 4 mm eventually merged over 24–48 h.

#### 2.3.3 Angle and Circular Printing

Figure 5 shows the impact on the printing resolution as the material deposited on overlapping points doubled, disrupting the uniformity of the layer height, while merging corners as the viscous hydrogel spilled and failed to retain its extruded form. This was rectified by increasing the viscosity of the hydrogel (samples 03 and 04 performed better than sample 02) or by increasing the speed of extrusion at that particular point, in order to deposit less material. However, the spilling of the material could not be completely eliminated.

#### 2.3.4 3D Layered Printing

Samples 03 and 04 demonstrated form‐ and layer‐retention properties, primarily due to their high viscosity, while sample 02 failed to maintain its printed form even when extruded at a high translational speed and low pressure. A maximum of 4 and 7 layers were deposited, with samples 03 and 04, respectively. This suggested the requirement of a bottom‐layer with a higher mechanical strength or strands with thicker diameters (or a material with an even higher viscosity), which could better hold in position the successive layers of print even prior to crosslinking.

#### 2.3.5 Flow Rate

The dissolved air introduced during the preparation of the hydrogel was observed to interrupt the constant rate of material flow, leading to unintended broken lines and curves during deposition, Figure 4a. We investigated several methods for degassing the gel such as agitation‐free mixing, exposing the mixed gel under a vacuum chamber, and ultrasonic agitation, none of which eliminated the trapped air from the hydrogel. Further, such techniques often adopted in the field of tissue engineering are not appropriate for such large‐scale applications where material is prepared and stored in sizable batches prior to extrusion.

This could be pursued in further work to improve the quality of the gel deposition. However, the presence of air bubbles within the hydrogel increases the rate of water absorption or evaporation, reducing the longevity of the hydrogel constructs postprint.^40^ Therefore, achieving a higher rate of homogeneity upon preparation of the hydrogel to achieve uniform flow rate might be a better solution instead of removing the air bubbles completely.

#### 2.3.6 Gelation Tests

To achieve a satisfactory printing resolution with reduced diffusion between adjacent layers (in *X*‐, *Y*‐, and *Z*‐directions), the printing time between two consecutive layers was delayed by 60 s during which the 3D printed scaffold was sprayed with 100 × 10^−3^ m calcium chloride solution and left under standard room temperature conditions to gain mechanical strength through crosslinking. This further impacted the deposition of additional layers, as the strands would not easily fuse together. This was mitigated by reducing the speed of deposition, allowing time for the strands to homogenously bind with the successive layers.

### 2.4 Design

**Figure 6:**
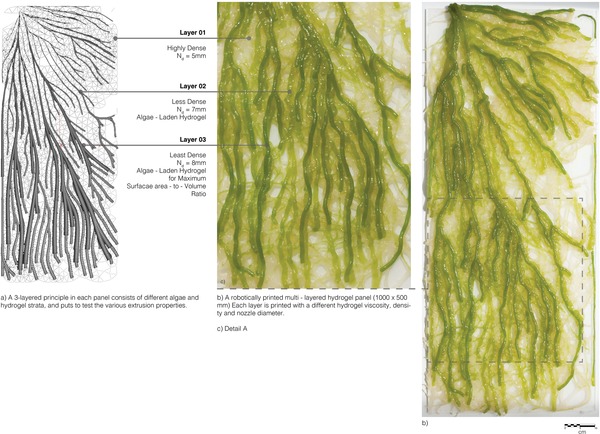
a–c) Robotically fabricated algae‐laden hydrogel panel (1000 × 500 mm).

**Figure** **6** shows the 1000 × 500 mm printed hydrogel panel. The pattern printed was aimed at demonstrating the multilayering of the extruded hydrogel with varying resolutions, augmenting the surface area within the panel potentially increasing the algae–nutrient contact within its surroundings. The branched geometry was developed keeping in mind the panels' potential application for bioremediation, in which water would flow over the algae‐laden hydrogel surface from one end (with a focused node) to the other (with an open and distributed array of branches). This further illustrated the possibility of successfully depositing higher concentrations of hydrogel layers toward the entry point which branched out into a more distributed layering toward the exit point. The design parameters allowing the scaling‐up of AM biological materials is exponential or gradient, instead of the rather linear approach. Naturally occurring structures demonstrate gradients that allow for the systematic change from the microstructure to a macroscopic scale. The branched design demonstrated here was generated to consist of layers with varying layer compositions along one axis, to seamlessly connect soft biocompatible layers with stiff surface depositions, much like a bone or a rock.^41^

The panel was divided into three horizontal layers, each varying in density and pattern, fabricated with different *N*_d_ and hydrogel viscosities. The bottom layer was densely printed up to 7 layers with a *N*_d_ of 5 mm and a high viscosity hydrogel (sample 03) in order to form a more mechanically stable grid‐like base capable of retaining the successive layers of hydrogel. It did not contain algae cells.

The top 2 successive layers containing immobilized algae cells were deposited in 6 and 3 layers, respectively. Layer 2, containing 89.2% water was deposited with *N*_d_ 5 mm and was denser than layer 3, following the bottom printed grid. Layer 3, containing 90–92.5% water was printed with *N*_d_ 7 mm, defining the geometry of the entire panel. The higher water percentage in layer 3 does not allow the deposition of more than 3 layers but permits optimum cellular growth due to increased photosynthetic activity through constant exchange of nutrients. Each layer was individually immobilized with CaCl_2_ during the printing process, after which the entire panel was immersed in a bath of CaCl_2_ for 30 min, allowing for additional crosslinking.

### 2.5 Limitations and Potential Applications

The focus of the present work was to demonstrate the possibility of fabricating large‐scale photosynthetic membranes from algae‐laden hydrogels. The initial work focused on developing a printing system which can be calibrated to extrude a range of rheologically characterized hydrogels suitable for cellular growth.

As hydrogels are made of 80–90% water, they are subject to high rates of evaporation. A comprehensive morphological understanding of the hydrogel in terms of its deformation and swelling caused via evaporation and absorption, respectively, postprinting along with their altering mechanical properties will need to be established. This could further allow us to integrate mechanical property prediction models within the design stage, to better tune the hierarchical gradations between the biological and the structural parts of the object.

To further improve the resolution of mechanical gradations achieved within the deposited membrane, it may be advantageous to develop an actuated mixing system at the nozzle using an auger screw, allowing to precisely mix the hydrogels with gradually varying mechanical properties prior to extrusion. This could be done by varying either the percentage of water or the concentration of monomers. In order to enhance mechanical and optical properties of the printed hydrogels, further material explorations should be conducted to explore alginate‐based hydrogels with additional rheology modifiers. This can be combined with an actuated mixing printer which could make possible the printing of such multimaterial homogenous hydrogels. A combination of extrusion and electrospinning techniques can also be explored to create macroscale vascular constructs with the aim of increasing the surface area‐to‐volume ratios within the resolution of the scaffold.^42^, ^43^, ^44^

The work presented here lays the foundations for applications such as the fabrication of photosynthetic skins or membranes to be applied in an engineering‐specific or in an architectural context. Potentially, these membranes could be applied on bioreceptive facades to improve water retention for self‐regulated biological growth on buildings and urban infrastructures.^7^, ^45^ Besides CO_2_ absorption through photosynthesis, algae cells are known to exhibit high sensitivity toward a range of heavy metal contaminants, allowing the possible use of these membranes as biosensors for environmental quality assessment.^46^ Microalgae wastewater treatment systems^47^ have also been developed, however they demand substantial amounts of land space to scale‐up the technology. The membranes presented here could be used within biotreatment systems as they allow confining the algal cells within a matrix while efficiently utilizing the volume of space available.^13^ Further, research is being conducted to explore the potentials of using hydrogels immobilized with algae and/or bacteria for the treatment of contaminated wastes.^48^ Besides single cell immobilization, efforts have also been made in the field of co‐immobilization to create efficient microsystems toward nutrient removal from wastewaters.^49^, ^50^, ^51^, ^52^

Immobilized microalgae for applications such as energy generation and wastewater treatment will require scalable systems. For example, hydrogen production^53^ or the use of algal biophotovoltaics to generate current^54^ within the built environment. Hence, this research introduces a design‐led platform with which biologically integrated living entities that harness the ability of microalgae for large‐scale applications relevant to the built environment can be developed.

## 3 Conclusion

Nature is known to display remarkable structural and environmental properties by hierarchically depositing the simplest of materials. However, research into additive manufacturing of similar structures from natural or synthetic polymers especially for applications in engineering or architecture is still in its infancy. This paper demonstrates the possibility of AM large‐scale algae‐laden hydrogel membranes via a multimaterial pneumatic extrusion system connected to the end effector of a robot arm. The rheological characterization of a range of hydrogels were performed and the relative printing parameters established, outlining an optimized process, enabling high repeatability and control. The printed membrane was deposited and cured at room temperature, eliminating the need for an external energy source besides the regular spraying of prints with CaCl_2_ for crosslinking.

A set of 8 alginate‐based hydrogel samples with varying polymers and water percentages were rheologically characterized to identify a window of operation, illustrating their suitability toward AM alongside their biocompatibility toward immobilized microalgae cells. Parametrically, the hydrogel sample was required to exhibit a low phase angle along with a dominant viscoelastic property, i.e., *G′* > *G″*, allowing the sample to flow through a nozzle under applied pressure however recovering its extruded geometry postextrusion. Alginate‐based hydrogels with rheology modifiers such as methylcellulose and carrageenan with water percentages between 80.6% and 92.5% were identified to be the most suitable. This is specific to the safe operating range of pressure set between 4 and 8.2 bars. All 8 samples were tested for their cellular compatibility, with alginate–methylcellulose hydrogel consisting of 85–90% water, showing the highest algae growth rate over a period of 21 days.

A large‐scale pneumatic multimaterial robotic extrusion platform was developed that could successfully deposit layer‐by‐layer the viscous hydrogel. Process optimization was achieved by varying the grade of hydrogel, nozzle diameters *N*_d_, flow rate *Q*, pressure *P*, and printing speed *S*. A particle simulation system was used to generate fibrous patterns with varying densities and thicknesses each relative to the hydrogel used for printing. Finally, a 1000 × 500 mm panel was fabricated from alginate–methylcellulose‐based hydrogel with varying water percentages with and without algae. The printed construct was maintained through regular hydration for a period of 21 days to observe cellular growth within the extruded hydrogel panels.

This work addressed the interplay of factors between material selection, design, and manufacturing of hydrogels on a large scale, providing a strategy for future applications in the areas of algal bioremediation, bioenergy and bioremediation.

## 4 Experimental Section

*Materials*: Sodium alginate and methylcellulose were purchased from Special Ingredients, UK. A cellulose nanofiber rheology modifier, Curran, CV 5000 at 7.73% solids was supplied by Cellucomp, UK. Laponite RD ms 16 was acquired from Conservation Resources UK. Ludox TM‐50 colloidal silica, 50 wt% suspension in H_2_O and Kappa‐carrageenan were purchased from Sigma‐Aldrich, UK.

*Hydrogel Formulation and Preparation*: Taking reference from a recent study that successfully fabricated photosynthetic algae‐laden hydrogel scaffolds on a microscale.^17^, ^18^ 2% w/v alginate solutions were prepared, and methylcellulose was added in a ratio of 3:1. Water was added to each of the samples to achieve a final percentage of 97%, 92.5%, 89.2%, and 80.6%. The solution was thoroughly stirred using an overhead mixer to obtain a homogenous paste and left at room temperature for 2 h to allow the swelling of methylcellulose.

To prepare alginate‐based composite hydrogels, sodium alginate (2% w/v) was prepared as before. Monomers – Carrageenan‐k, Curran CV 5000, Laponite RD, and Ludox TM‐50 were added in a ratio of 3:1 with alginate and thoroughly stirred to obtain a homogenous plotting paste as per Table 1.

In all experiments, CaCl_2_ (100 × 10^−3^ m) was used for crosslinking within 10 min of printing. The fabrication process was conducted at room temperature.

*Rheological Characterization*: Rheological characterization was performed on all hydrogel samples using a Malvern Kinexus Pro+ rotational rheometer (Worcestershire, UK). The test geometry was a 50 mm diameter plate, with a 1 mm zero gap and temperature was maintained at 25 °C. Hydrogels were stored at room temperature until a 2 mL sample was removed and dispensed onto the rheometer plate.

Rheological characterization was performed in three steps. First, amplitude sweep with strain values from 0.01% to 10% and 10 to 100% were applied on each sample separately to determine their respective limits of LVE behavior. Frequency sweeps with frequency 0.1–10 Hz and strain value 0.5% and 0.1% were then conducted on each sample to determine the linear equilibrium shear modulus plateau of the hydrogel. This further determined the lowest frequency limit at which the material behavior changed from gel to solid. Flow curve test based on shear stress and shear strain (shear rate ranging from 0.1 to 100 s^−1^) was conducted on each sample to reaffirm their shear thinning behavior. Extrusion pressures mentioned in Table 1, were recorded for each of the samples with a volume of 150 mL when extruded through a nozzle diameter *N*_d_ of 5 mm.

The accuracy of the amplitude–oscillatory shear moduli measurements depended on the torque magnitude generated by the deformation and by the instruments ability to resolve the phase angle between the strain and stress waves. Each sample was tested 3 times to reassert the measurements reproducibility and repeatability. For certain samples, with notably high viscosity that depicted noisy and irregular readings on the rheometer, multiple readings with separate samples were taken in order to eliminate the deformation of the hydrogel under stress.

*Preparation of Algae‐Laden Hydrogels for Printing—Cell Culture: C. sorokiniana* (CCAP 211/8K) was obtained from Culture Collection of Algae and Protozoa (Oban, Scotland, UK). Cultures were prepared by inoculating cells in modified tris‐acetate phosphate (TAP) media (Kropat et al.)^55^ and incubated in an illuminated incubator at 25 °C until cells reached exponential phase. 10% of water was substituted for algal cell culture during the preparation of hydrogels prior to printing.

*Preparation of Algae‐Laden Hydrogels for Printing—Biocompatibility and Shear Analysis*: Each of the 8 alginate‐based hydrogel samples were prepared with algae cell culture as described previously. The samples were then transferred onto petri dishes and saturated in a 100 × 10^−3^ m CaCl_2_ solution for 5–15 min to allow for crosslinking of the hydrogel. Petri dishes were covered and sealed with Parafilm. Each petri dish was supplied with 5 mL solution of acetate‐free TAP media every 7 days, in order to maintain the growth of algal cells and replenish water lost through evaporation. Growth of algae on a macroscale was photographically recorded over a period of 21 days (Figure 3).

The ultra shear device (USD) was described previously^56^, ^57^, ^58^, ^59^ as a method to assess the potential for shear force to induce cell breakage. Briefly, 20 mL samples were exposed to shear stress using a rotating shear device with a stainless‐steel chamber of 50 mm diameter and a height of 10 mm along with a rotating disk of 40 mm diameter and 1 mm thickness. The maximum speed in the shear device (18 000 rpm) was held for 20 s, corresponding to shear at 2.63 × 106 W kg^−1^. This was chosen to represent a high shear environment, such as that experienced in the nozzle during material deposition.^60^ Experiments were performed in triplicates alongside an unsheared control experiment. 10 mL of sheared algae solution was transferred into flasks with 30 mL of TAP media. All flasks were observed in an incubator (22 °C, 100 rpm) for 7 days and optical density (OD) readings at 750 nm were recorded on day 0 (immediately after shearing), day 3, day 4, day 5, day 6, and day 7.

**Figure 7:**
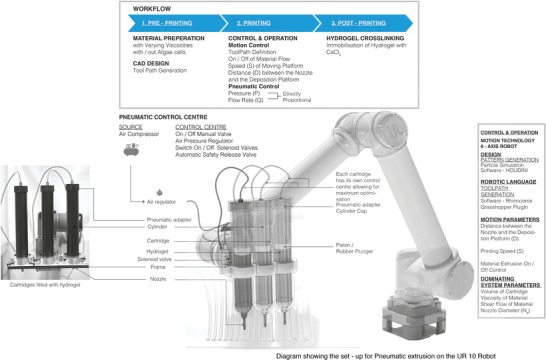
Schematic depicting the large‐scale bioprinting process – starting from the preparation of the material to the pneumatic extrusion via cartridges attached on the robotic arm, followed by alginate–calcium immobilization by immersing the print in 100 × 10^−3^ m calcium chloride solution.

*Robotic Fabrication—Hardware and Printing Setup*: A small industrial 6‐axis robot arm UR 10 (Universal Robot) with a working radius of 1300 mm was used to position a custom deposition head attached to the robot's end‐effector to print single and multilayer hydrogel structures. **Figure** **7** shows a general arrangement of the printing system's components. Initial tests were conducted with a custom‐built single cartridge extruder to characterize the printing parameters, this was upgraded to a 3‐cartridge system for the fabrication of the large panel. Briefly, the system was set up as follows.

Mounted on the 6‐axis robotic arm this 12 oz cartridge extruder was controlled with a 3‐way, 2‐position solenoid valve that regulated the mechanically driven airflow from the main pressure gauge. The clear plastic dispensing cartridges with rubber plungers made of thick walls, wide flanges and designed for use under high pressures were purchased from Adhesive Dispensing, UK. The internal bore of the cartridges was kept at “zero draft” (no taper), allowing the rubber piston to slide through smoothly under pressure, maintaining a constant dispensing flow. These cartridges were further secured within hard plastic casings with a screw top that secured the airflow (also purchased from Adhesive Dispensing, UK). Custom designed mountable end effector components were built from aluminum sheets cut with a numeric control water jet‐machine to hold the cartridge(s) in position on the robotic arm. The pneumatic circuitry was built with flow‐regulating solenoid valves, air pressure gauges, and plastic tubing of 4 mm internal diameter at 10‐PSI max.

The deposition cartridges were filled with the hydrogel formulation and securely loaded into the plastic casings. Each cartridge was suffixed with a plastic nozzle tip with diameters that could be varied depending on the design to be printed from 2 to 7 mm. The resolution of the print and the speed of printing was dependent on the flow rate of extrusion, which in turn was a function of the extrusion pressure directly proportional to the viscosity of the hydrogel. The pressure was maintained within 4–8.2 bars. At the start of the printing sessions, the flow rate was measured using a gravimetric‐time method found to be nominally 12 g min^−1^ through a *Ø* 3 mm nozzle at the maximum pressure of 8.2 bars.

*Robotic Fabrication—Software*: The mechanics of the robot's printing movement – speed, distance from deposition platform *d*, and points of extrusion were generated using the Rhino (2013, Rhinoceros, Robert McNeel and Associates, USA) plug‐ins Grasshopper and Robots.^35^

*Computational Design*: A procedural particle simulation technique was used within Houdini software (16.0, Side Effects Software). The Houdini node for finding the shortest path between a preselected set of points within a defined point cloud was used to calculate optimum paths between the start and end points, generating a set of polygonal curves. The “path cost” node and the “minimum adjacency cost” node were used equivalent to the minimum gap required between adjacent lines in order to prevent diffusion of the hydrogel, optimizing each segment length creating a gradient pattern to test the resolution of the printing system. The curves generated varied for hydrogels with different rheological properties and were determined via initial printing tests. A series of membrane configurations were simulated and printed to develop 3D structures.

## Conflict of Interest

The authors declare no conflict of interest.

## Supporting information

Supporting Information
