# Supplementary material for: Robotic Extrusion of Algae‐Laden Hydrogels for Large‐Scale Applications
Source: Glob Chall. 2019 Nov 11;4(1):1900064. doi: 10.1002/gch2.201900064 (PMC6957016; doi:10.1002/gch2.201900064)
Supplement: Supplementary file 1 — Supporting Information [file GCH2-4-1900064-s001.pdf]

Copyright WILEY-VCH Verlag GmbH & Co. KGaA, 69469 Weinheim, Germany, 2019.

Supporting Information

for *Global Challenges,* DOI: 10.1002/gch2.201900064

Robotic Extrusion of Algae-Laden Hydrogels for Large-Scale Applications

Shneel Malik, Julie Hagopian, Sanika Mohite, Cao Lintong, Laura Stoffels, Sofoklis Giannakopoulos, Richard Beckett, Christopher Leung, Javier Ruiz, Marcos Cruz, and Brenda Parker**

Supporting Information

Robotic Extrusion of Algae-Laden Hydrogels for Large Scale Applications

Shneel Malik, Julie Hagopian, Sanika Mohite, Cao Lintong, Laura Stoffels, Sofoklis Giannakopolous, Richard Beckett, Christopher Leung, Javier Ruiz, Marcos Cruz, Brenda M Parker**

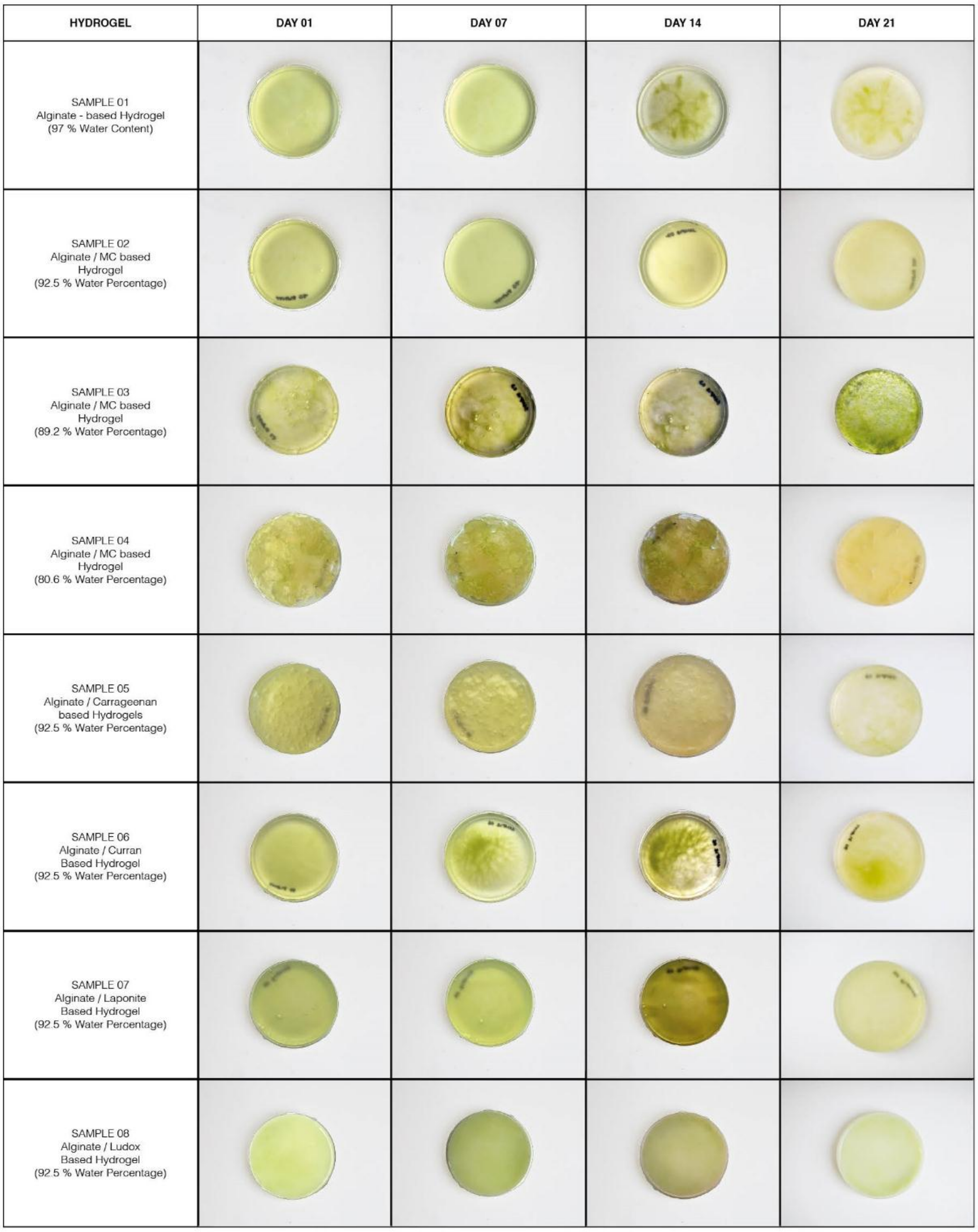

Figure S1 Cellular viability tests are conducted on a macroscale. Petri dishes were supplied with 5mL TAP media every 7 days in order to maintain the growth of algae cells.
